# Ethical concerns in suicide research: thematic analysis of the views of human research ethics committees in Australia

**DOI:** 10.1186/s12910-021-00609-3

**Published:** 2021-04-07

**Authors:** Emma Barnard, Georgia Dempster, Karolina Krysinska, Lennart Reifels, Jo Robinson, Jane Pirkis, Karl Andriessen

**Affiliations:** 1 Centre for Health Equity, Melbourne School of Population and Global Health, The University of Melbourne, Parkville, VIC 3010 Australia; 2 Centre for Mental Health, Melbourne School of Population and Global Health, The University of Melbourne, Parkville, VIC 3010 Australia; 3 Orygen, The National Centre of Excellence in Youth Mental Health, Parkville, VIC 3052 Australia

**Keywords:** Ethical review, Ethics, IRB, Research, Research ethics committee, Suicide, Suicide prevention

## Abstract

**Background:**

Suicide research aims to contribute to a better understanding of suicidal behaviour and its prevention. However, there are many ethical challenges in this research field, for example, regarding consent and potential risks to participants. While studies to-date have focused on the perspective of the researchers, this study aimed to investigate the views and experiences of members of Human Research Ethics Committees (HRECs) in dealing with suicide-related study applications.

**Methods:**

This qualitative study entailed a thematic analysis using an inductive approach. We conducted semi-structured interviews with a purposive sample (N = 15) of HREC Chairs or their delegates from Australian research-intensive universities. The interview guide included questions regarding the ethical concerns and challenges in suicide-related research raised by HREC members, how they dealt with those challenges and what advice they could give to researchers.

**Results:**

The analysis identified four main themes: (1) HREC members’ experiences of reviewing suicide-related study applications, (2) HREC members’ perceptions of suicide, suicide research, and study participants, (3) Complexity in HREC members’ decision-making processes, and (4) HREC members’ relationships with researchers.

**Conclusions:**

Reliance on ethical guidelines and dialogue with researchers are crucial in the assessment of suicide-related study applications. Both researchers and HREC members may benefit from guidance and resources on how to conduct ethically sound suicide-related studies. Developing working relationships will be likely to help HRECs to facilitate high quality, ethical suicide-related research and researchers to conduct such research.

**Supplementary Information:**

The online version contains supplementary material available at 10.1186/s12910-021-00609-3.

## Background

Suicide is a persistent public health problem. Worldwide, approximately 800,000 people (10.6 per 100,000 people) die by suicide annually, and for every suicide, there are more than 20 suicide attempts, which has a huge impact on families, caregivers, and communities at-large [1]. Sadly, over the last decade, the suicide rate in several countries has increased. For example, in Australia the rate increased from 10.9 in 2008 to 12.2 in 2018 [2]. Such increases have fuelled public concerns and calls for more suicide-related research to optimize prevention approaches [3].

While high quality research may inform effective practices and policies in suicide prevention, research in this field faces ethical challenges. These include participant safety and informed consent and whether individuals who are perceived to be at an increased risk of suicide should be asked to participate in intervention studies [4, 5]. On the one hand, there are concerns that including at-risk participants in research may cause them distress and run counter to researchers’ duty of care. On the other hand, there is the view that excluding them will preclude the assessment of effectiveness of interventions for those most in need [4, 6]. Balancing these views requires an in-depth examination of who holds them and why they are held. Of particular relevance are the experiences of researchers who successfully obtain ethical approval for suicide-related studies, and the experiences of research ethics committee members reviewing such studies. Only a few studies have investigated the experiences of these two groups, and most of these have focused on researchers.

Lakeman and Fitzgerald conducted a survey with a sample of 28 researchers, mostly located in the UK and the USA [7]. Few researchers in this study had experienced major problems in obtaining ethics approval, possibly due to their careful anticipation of concerns related to accessing the study population, maintenance of confidentiality, and responsibility of care for participants. However, some researchers reported that they had encountered resistance from their research ethics committee when seeking approval for suicide-related studies. Researchers in other fields have also reported having experienced an over-protective or even paternalistic attitude of research ethics committees concerning sensitive research [8] and have highlighted tensions between researchers and their committees due to a mismatch of expectations and confusion about each other’s roles and responsibilities [9, 10].

Recently, our team conducted two surveys regarding researchers’ experiences in obtaining ethics approval for suicide-related studies in Australia and internationally [11, 12]. In both surveys, respondents reported that research ethics committees were mostly concerned about potential harm to participants and researchers’ duty of care, while there were only occasional concerns regarding researchers’ competency and safety. Most respondents in both surveys modified their ethics application and/or consulted with their research ethics committee to address the concerns raised and reported that the committees’ feedback had either a positive impact or no impact on their planned study. The findings suggested that researchers in this field can anticipate potential concerns and communicate effectively with their research ethics committees.

To the best of our knowledge, to-date only one study has looked at the experiences of members of research ethics committees regarding dealing with suicide-related study applications [16], although there have been studies on how research ethics committees deal with other sensitive topics [10, 13–15]. In that study, Lakeman and Fitzgerald [16] surveyed 125 members of international research ethics committees, mostly based in the UK and Canada. Respondents reported potential harm to research participants, responsibility of the researcher to participants, and participants’ competency and consent as the major concerns. Additionally, some respondents perceived their research ethics committees as paternalistic [16].

This study was designed to take a further, more in-depth view of research ethics committee members’ experiences in dealing with suicide-related study applications and aimed to investigate their experiences in assessing and deciding about such study applications. More specifically, the study explored the following research questions: (1) what issues are important for members of research ethics committees when evaluating suicide-related study applications? (2) What are the key ethical challenges? (3) How do members of research ethics committees deal with those challenges?, and (4) What kind of advice can they give to researchers in this field?

## Methods

### Study design

This was a qualitative interview study utilising a thematic analysis approach. We report the findings based on the Consolidated Criteria for Reporting Qualitative Studies (COREQ) checklist [17] (see Additional file 1). The Human Research Ethics Committee of The University of Melbourne approved the study on May 28, 2019 (ID1852648.3). The semi-structured interviews were conducted between June and November 2019.

### Sampling

We recruited a purposive sample of members of Human Research Ethics Committees (HRECs) from Australian research-intensive universities. HREC is the standard name for research ethics committees in Australia, otherwise known as Institutional Review Boards (IRBs) in the United States and Research Ethics Committees (RECs) in the United Kingdom. In Australia, the National Health and Medical Research Council (NHMRC) National Statement on Ethical Conduct in Research [18] requires that HRECs comprise at least eight members. Wherever practicable, there should be equal numbers of women and men, one third of members should be from outside the institution, there should be an HREC Chair, two lay people, one person with current experience in professional health or allied healthcare, a pastoral care person, a lawyer and two people who are active researchers with relevant experience [18].

In this article, “respondents” are the HREC members who participated in our study, and “participants” are those who participated in the studies reviewed by the respondents’ HREC or in research in general. Eligible respondents were (a) the Chair of a formally constituted HREC at a research-intensive Australian university or, (b) a nominated delegate of an HREC Chair. Chairpersons were identified as suitable respondents due to their knowledge of and experience in how HRECs function and make decisions. Eligible respondents had been a HREC Chair or member for at least two years, an inclusion criterion that has successfully been used in other research [9], and had reviewed at least one suicide-related study application. According to the literature, the required sample size to answer the research questions depends on the complexity of the research questions, the homogeneity of the study population, and the interview structure [19–21]. A total of six to twelve interviews may suffice for a highly experienced research team [21]. Based on this literature and our experience, we estimated the required sample size between 12 and 15 interviews.

We generated a list of the 20 most research-intensive universities in Australia, based on the Times Higher Education rankings. Once recruitment was underway, we expanded this to the top 35 universities to meet the target number of respondents. Between June and September 2019, we emailed 44 HRECs at these 35 universities to ascertain interest in participating. A maximum of two reminders were sent, and 30 HRECs replied to the invitation. Of these, 15 agreed to participate and 15 did not meet eligibility due to either having a Chair who was new to their role or not having reviewed any suicide-related study applications. We did not receive replies from 14 of the HRECs contacted. There were no reimbursements offered to respondents. Table 1 summarises the respondents’ characteristics that were collected during the interview process.

**Table 1:** Respondents’ characteristics (N = 15)

	n (%)
Gender	
Female	9 (60.0%)
Male	6 (40.0%)
Location (state/territory)	
New South Wales, Victoria	8 (53.3%)
Australian Capital Territory, Queensland, South Australia, Tasmania, Western Australia	7 (46.8%)
Years of experience in HREC	
< 5 years	2 (13.3%)
5–10 years	4 (26.7%)
> 10 years	9 (60.0%)

Respondents came from universities across all jurisdictions in Australia, except the Northern Territory. Most respondents were highly experienced as they reported an estimated HREC membership length ranging from three to over 20 years (*M* = 10.21, *SD* = 6.82). Fourteen respondents were academic researchers, and one respondent fulfilled the pastoral care role of their HREC. In addition to their university HREC roles, respondents reported membership of other types of ethics committees, including Government, private, animal, and health service/hospital ethics committees. Thus, some respondents sat on multiple committees concurrently as researchers, pastoral care members, and as lay people (for example on a health service committee).

Two respondents indicated they reviewed more than six study applications per year where suicidal behaviour was the primary research focus and estimated they had reviewed more than 30 such applications in the last five years. Eleven respondents reported reviewing either one or two suicide-related applications per year. The remaining two respondents had reviewed suicide-related applications in the context of broader mental and other health project applications, which included questions about suicidal ideation or behaviour.

### Procedure

Two researchers (EB—the lead researcher—and KA, both experienced qualitative researchers) drafted the interview guide (see Additional file 2), which was tested in a mock interview and approved by the research team. EB conducted the semi-structured interviews, which allowed exploration of responses given by respondents. Interviews were conducted in English and audio recorded. Consent to participate in the study was obtained for all respondents. Eleven respondents returned their written consent forms via email prior to or at the time of interview. Four respondents who did not return their written consent forms via email, indicated via email that they consented to participate in the context of organising their interview and subsequently provided verbal consent which was recorded at the time of interview. All respondents were asked at the time of interview if they were still willing and consenting to be interviewed for the study, irrespective of previously having provided written consent. These procedures met the criteria for consent as approved by our research ethics committee.

Respondents determined how they wanted to conduct the interview. Twelve interviews were conducted remotely via telephone and one via videoconferencing, all from a private office. The two other interviews were held in-person on a university campus in Victoria, Australia. Interviews ranged in length from 26 to 55 min (*M* = 38.06, *SD* = 9.84). The interviewer recorded field notes after the interviews, and during listening to the audio recordings or reading the transcripts. Transcripts were created with the assistance of NVivo transcription software [22]. EB checked the transcripts for accuracy and deidentified them prior to analysis.

## Research team and reflexivity

EB kept a research journal for the duration of the study. KA and JP, both experienced researchers, supervised EB during the data collection process. The whole research team met on a regular basis to discuss the progress of the data collection, and to reflect on the content of the interviews and the interactions between interviewer and respondents. None of the researchers had a prior relationship with respondents. One researcher (GD) may have known some respondents due to her previous role as an HREC executive officer. To minimise bias, GD was not involved in recruitment or data collection, and was not privy to the identified list of respondents.

### Analysis

We conducted a thematic analysis using an inductive approach. Thematic analysis aims to identify, organise and interpret patterns of meaning or themes across a qualitative data set [23]. Our analysis was guided by the six-steps framework of Braun and Clarke [24], and most closely resembles what they conceive of as “codebook thematic analysis” [25]. It involved an iterative process of reading and rereading the data, producing initial codes, grouping codes in potential themes, reviewing and refining potential themes against the data, which were then ultimately conceptualised as domain summaries. Initially, three researchers (EB, GD and KA) analysed the same three transcripts to agree on the codebook [25]. Subsequently, EB and GD coded the remaining transcripts. Any disagreement was resolved through discussion involving the third researcher (KA). This process allowed us to determine the coding structure and to ensure our approach was valid. EB, GD and KA further analysed the data, grouped codes and refined themes, identified subthemes, and finally domain summaries. The last interviews did not yield new information. Hence, we are confident that the study collected enough data to answer the research questions.

## Results

The analysis resulted in four main themes: i) HREC experiences of reviewing suicide-related study applications, ii) HREC perceptions of suicide, suicide research, and study participants, iii) Complexity in HREC decision-making processes, and iv) HREC relationships with researchers.

### Theme 1: HREC experiences of reviewing suicide-related study applications

Respondents were asked to recount their experience reviewing suicide-related ethics applications. This was an open question, where no assumption was made about the specific types of suicide research that respondents had reviewed. Respondents spoke to their experience of reviewing a range of different types of qualitative, quantitative and mixed-methods research involving suicide and/or self-harm, including primary prevention research involving humans, cross-sectional survey-based research, and observational studies.

Notably, it was more common for their HRECs to encounter suicide as an incidental concern in broader mental and other health-related study applications than when specifically reviewing suicide-related study applications. Suicide as an incidental issue in research was defined as when researchers might ask about suicidality as part of a battery of psychometric tests, or where study participants would meet criteria for severe depression. Here, the potential for participant distress was as much a concern for HRECs as in applications that addressed suicide specifically, as stated by this respondent*: *“*It's certainly something we do get very concerned about. We know what level of distress people may experience and what the potential is for an extreme reaction.*” [Respondent #1].

For all study types, there was consensus that suicide as a topic was not a reason in itself for requesting changes to study applications. The respondents saw the role of HRECs as facilitative; however, they were aware that research ethics committees can be perceived as obstructive: “*Our goal is for the ethical conduct and effective and beneficent research, not to prevent or hinder or stop or obstruct research. Although that might be how some individual might experience it.*” [Respondent #6] Respondents also considered the HRECs’ role as active in getting projects approved and spoke at length about the processes they used for this to occur, both in the formal review and externally, such as meeting with researchers, or providing education to graduate student researchers.

#### Characteristics of a strong application

When respondents reflected on how HRECs reviewed suicide-related study applications, they spoke to three subthemes that determined a strong application. HREC decisions were dependent on the merit and integrity of the research being proposed, the relevant expertise of the researcher, and the HREC’s perception of potential risks to participants coupled with proposed mitigating strategies. As one respondent stated: “*It's all about managing the risk to participants and doing something that has merit and integrity.*” [Respondent #12] Ethics applications that did not adequately address these concerns would likely be sent back to researchers.

#### Research merit and integrity

First and foremost, HRECs wanted researchers to clearly demonstrate the research merit and integrity of their project. They wanted to see applications where the proposed research was clearly justified by its potential benefit and designed employing research methods that were commensurate with the study aims and objectives. When discussing research merit and integrity issues they had seen reviewing suicide-related ethics applications, one respondent stated: “*That's kind of a tough one.*” [Respondent #8] The example provided was when their HREC found a problem with the proposed research question. These types of problems were described as being difficult to resolve because research merit and integrity are fundamental to ethical research design. Respondents agreed that where researchers had not clearly articulated the research merit and integrity of the proposed research, HRECs would typically take issue with the application.

#### Relevant expertise

Secondly, HRECs wanted to see expertise around suicide built into research projects when it was lacking. Respondents described valuing research experience itself, in addition to familiarity with suicide as a research topic. Experienced research teams were often advised to consult with content experts to get comprehensive advice about their proposed research, as one respondent noted: “*We do make recommendations all the time about … research teams trying to bring on other collaborators to address those gaps.*” [Respondent #1] In the case of novice researchers, HRECs would recommend appropriate supervision by more senior colleagues. The professional background and experience of novice or junior researchers was also a consideration for HRECs. For example, one respondent spoke about a complex suicide-related graduate student research project. This application was eventually granted ethical approval due in part to the student’s professional background as a mental health nurse, combined with their supervisor having many years’ experience researching vulnerable population groups. HRECs wanted to see researchers with all levels of experience consider their relevant expertise and identify opportunities to enhance it when doing so would benefit the research project.

#### Strategies to mitigate risks to participants

Thirdly, respondents saw HRECs’ primary role as protecting the people participating in suicide-related research. When asked about ‘risk’ respondents broadly interpreted this as risks to participants in the first instance. HRECs were concerned with risks to participants irrespective of the study design. For example, respondents reported being equally as concerned about participants experiencing distress in a face-to-face research project, as they were about participants who might be completing an anonymous survey with no direct contact with researchers. Additionally, balancing potential risks to participants with the potential benefits of the research was described as the key priority for HRECs, as one respondent said: “*We definitely understand the important need to do this research, but our primary goal is to protect the participants from harm directly related to the research.*” [Respondent #7].

Respondents reported HRECs’ concerns about how to manage distress if a study participant indicated that they were suicidal. Respondents wanted to see researchers provide specific, worked examples of how they would protect research participants, such as protocols for researchers to monitor and manage potential distress in participants involved in suicide related research: “*Any kind of research would have to manage any possible risks … for example people exhibiting distress, emotional or mental distress, they would have to have protocols of response if a person declared suicidal behaviour or suicidal ideation.*” [Respondent #6] HRECs wanted to see evidence that researchers had thought about participant risk. Examples were provided, such as strong management plans addressing potential risks to participants, and clearly articulated protocols for addressing participant distress at all stages of a research project.

### Theme 2: HREC perceptions of suicide, suicide research, and study participants

Throughout the interviews, respondents shared their personal views on suicide, both its causes and prevention. They also addressed features of suicide research that could cause concern in HREC members. In this context, respondents stressed participant safety as a primary concern of HREC members.

#### Perceptions of suicide

While we did not directly ask about respondents’ knowledge of suicide, several respondents offered their own opinions about what causes suicide, who is most at risk, what increases risk, or how suicidal people present. There was a perception among respondents that lay members of HRECs are more concerned about suicide as a topic than academic committee members: “*Ethics committee members particularly the external members … tend to get very, very anxious about the mere fact that you're asking people about suicide.*” [Respondent #2].

Respondents also acknowledged that HREC members’ perceptions of suicide were likely to be different if they were clinically trained, compared to members without such training. One respondent indicated: “*I’m a clinical psychologist myself … We explain clearly that it's okay to ask about suicide, that we should be asking about suicide so we don't really shy away from those things being included in a research project.*” [Respondent #15] A core value of HRECs expressed by respondents was that the variety of backgrounds and views of HREC members helped to ensure that applications were reviewed with the broadest possible outlook, and therefore helped researchers to think about suicide and risk outside of their own discipline.

#### Perceptions of suicide research

We asked respondents if there were any study design issues that worried HRECs in the context of suicide-related research, such as the overall research design, data collection techniques, or participant recruitment methods. Respondents were able to recount particular examples where study applications had not adequately addressed potential risks to participants and resoundingly made the point that HRECs would want to see a risk management plan, regardless of the study design. Further, all study designs were ‘good’ as long as the HREC was satisfied that the project had research merit and integrity. Respondents were clear that it was not the role of HRECs to attribute the assessment of risk to the study design or to discourage research that might be considered as ‘high risk’, as one respondent declared: “*We've encouraged [researchers] to do higher risk research because we think it's really important. Don't be frightened of doing research that some people are going to consider high risk.*” [Respondent #8] Here, the participant pointed to smaller qualitative interview studies as an example where their committee would not be concerned about the research design as long as it was robust and appropriate risk mitigation strategies were articulated.

Additionally, respondents were unanimous of the view that it is important for HRECs to understand suicide as a major health and social issue. They were cognisant that their role was to weigh the potential benefits of ‘risk’ or ‘high-risk’ research, as one respondent commented: “*It's really important that we know this stuff. We've got these particular suicide rates … we don't understand why these things are happening and if we spend a lot of time holding this up, what kind of knowledge are we preventing?*” [Respondent #13].

#### Perceptions of study participants

Many respondents were extremely concerned with the welfare and safety of participants in suicide-related research: “*By their very nature HRECs are created to worry about participants.*” [Respondent #2] Respondents expressed concern about labelling groups of people as vulnerable, but at the same time were able to extensively list participant groups that might cause concern when reviewing an ethics application. This included children and young people, older adults, people with mental health problems, people with cognitive or intellectual disabilities, Aboriginal and Torres Strait Islander people, and lesbian, gay, bisexual, transgender, queer or questioning, intersex and asexual (LGBTIAQ +) populations. Respondents wanted to see researchers think about extra measures to mitigate risk or manage potential distress in particular groups of study participants and in certain settings, such as school-based research: “*You know if it's at a school, it's going to be very carefully scrutinized. I mean nothing is impossible, but it would trigger a whole lot of extra as you say worry or concern*.” [Respondent #15] Another respondent raised that their HREC was more comfortable about studies where people with personal experience of suicidal behaviour volunteer to participate, as opposed to studies where participants might be asked about suicide in a battery of test questions. Here the decision of a participant to specifically opt-in to a suicide-related study was less concerning than a situation where a participant might be ‘surprised’ by a question about suicide or self-harm that they perhaps were not anticipating.

### Theme 3: Complexity in HREC decision-making processes

Respondents discussed how their HRECs went about making decisions in relation to suicide-related studies and to frame feedback to applicants. Two key concepts dominated this discussion: how committees use the NHMRC National Statement on Ethical Conduct in Research [18], and their personal feelings and opinions about suicide and suicide-related research.

#### The use of the NHMRC National Statement

One respondent noted that the latest version of the National Statement [18] makes it easier for HRECs to ask for more detail from researchers, and described a new utility to the most recent version of the guidelines: “*It gives the committee much more scope to ask questions … now it's codified and we can say no look, the National Statement says we have to ask this … you've got to actually provide a ‘How will you know that participant is distressed or what’s your training around doing interviews?’… Whereas before we'd say to researchers, ‘So what are you going to do if you get the interviewee gets upset?’*” [Respondent #9].

However, several respondents described using the National Statement [18] as both a general framework and a specific guide for decision-making about suicide-related study applications. Respondents reported that their HRECs placed different emphases on its use in the decision-making process. For example, one respondent spoke about the National Statement [18] in terms of the HRECs accountability in reviewing ethics applications, describing their HREC as having an: “*obligation to researchers to make sure that whatever they do complies with the National Statement [and] an organisational responsibility to make sure that the organisation's researchers are compliant with the National Statement.*” [Respondent #12] However, another respondent described using the National Statement more as a general guideline: “*There's not often, apart from the waiver [of consent] issue there's not often specific chapter and verse that we'll quote back to the researchers about ‘you've satisfied certain aspects of the National Statement from our perspective’… But generally, the National Statement is a more general framework for us.*” [Respondent #10] Alluding to the feedback HRECs provide to researchers, this respondent singled out waiving consent as an issue to be guided wholly by the National Statement [18], while suggesting that other issues did not require the same attention.

#### Personal feelings and opinions

Respondents described the reflexive and reflective nature of assessing ethics applications, and how personal experiences and opinions did play a role in decision-making. Some respondents spoke about the usefulness of the National Statement [18] as a means to guide and structure HREC discussions around (especially non-academic) HREC members’ feelings about suicide. It was reasonably common for HREC members to share personal feelings, opinions, and experiences as part of the discussion about suicide and other sensitive research topics: “*Some of the committee provided personal experiences and how they've come through those as part of the balancing or consideration of the act, of the activity.*” [Respondent #3] Additionally, respondents described allowing for the discussion of personal feelings and opinions as part of the overall ethics review process: “*So, you know we often bring our own … feelings into it but at the same time we also are very conscious of the fact that just because we think something might be sensitive doesn't mean the rest of the world does.*” [Respondent #13].

### Theme 4: HREC relationships with researchers

Most respondents stated that good relationships between HRECs and researchers were essential to facilitate the research process, as this comment illustrates: “*Ethics is about relationship building and we want people to experience the ethics committee as something that facilitates their research and protects them as well, not just something that is obstructionist.*” [Respondent #13] Respondents spoke at length about the strategies they employed to develop and cultivate good relationships with researchers, both in terms of the interpersonal interactions with researchers and when framing feedback to researchers about their ethics applications. It was more common to meet individually with researchers to discuss applications that were not up to a standard to be approved, than for researchers to present to the whole HREC. The process of discussing the research with an HREC member can help to clarify an application: “*Sometimes we do sit down with researchers and go through things because what might seem really clear to them may not be so clear to someone else.*” [Respondent #5] Individual attention from the HREC Chair was noted as a way to clear up any misinterpretation on either side, to facilitate amendments and to get ethics applications approved as efficiently as possible.

Several respondents explicitly stated that the goal was to get applications approved, which necessitated clear and transparent communication from HRECs to researchers: “*It's not a mystery. It's not the researchers’ job to guess what's in the HREC Chair’s head or the committee black box, or whatever you call it. This should be quite transparent*.” [Respondent #12] One respondent described this approach as ‘making suggestions’ to researchers rather than ‘prescribing changes’. Strategies respondents employed to facilitate adaptive communication were to explain components of the NHMRC National Statement [18] to researchers, to provide examples about how to address and mitigate risk, to ensure the tone of written feedback was supportive and not combative, and to work closely with HREC secretariats who often have more direct contact with researchers.

Most respondents reported that when researchers had more experience with suicide as a research topic, the quality of the applications tended to be better. The relationships that developed over time between HRECs and researchers facilitated an improvement in the quality of ethics applications, as the researchers learned to better manage risk within their study design and better articulate the balance between risk and potential benefit of the research. One respondent highlighted that HRECs learn as much from researchers as researchers learn from HRECs and noted the reciprocity inherent in the relationship: “*As a committee we recognise that research is at the cutting edge of everything … we understand that these can be the first times these kinds of methods or techniques or whatever have been used, particularly if they apply to suicide and mental health. And so, we're trying to work through the issues and troubleshoot the issues at the same time as the researchers.*” [Respondent #7].

Respondents reported encountering frictions or tensions with researchers. They stated that when HRECs raised issues of risk (as framed by the NHMRC National Statement [18]), sometimes researchers misinterpreted the potential risks and benefits involved in their research or argued that their research approach was not risky, as one respondent noted: “*[Researchers] don't recognise, they think oh this is what I intend and that's not risky … so it is a process of educating people to understand what is meant by risk. And I mean generally, researchers have good intent. So, there is usually a very co-operative response.*” [Respondent #6] Largely, such issues were reported when researchers had little experience. This occurred with novice researchers, but also with experienced researchers new to suicide as a research topic. Nonetheless, respondents noted that friction with experienced suicide researchers was rare: “*You get a lot of fighting or pushback on the ethics committee with some researchers but those that are dealing with suicide, we haven't really had it. Probably because they're working in an area where they have to think about the ethical issues.*” [Respondent #15].

## Discussion

This study aimed to explore the experiences of Australian HRECs in dealing with suicide-related study applications. In terms of the issues deemed important when assessing such applications, respondents discussed the key ethical challenges and how these were managed. Also, by responding to the interview they were able to advise researchers in this field. Importantly, respondents clearly stated that their HRECs want to approve robust suicide-related studies. Respondents confirmed that the HRECs were committed to facilitating research that seeks to better understand suicide and to advance knowledge about its prevention. This contrasts with prior research which had found that some researchers had experienced obstruction and resistance from research ethics committees regarding undertaking suicide-related research [7]. However, respondents in this study articulated a clear commitment to facilitating suicide-related research, while acknowledging a need to ensure that it is conducted as safely as possible to protect study participants from potential harm.

A key finding was that HRECs were more likely to encounter ethical issues in the context of broader mental health studies than in suicide-specific studies. Respondents stated that they assessed many more broader study applications than applications that specifically focus on suicide and its prevention. Respondents indicated that in these situations, concerns with study applications were more likely to occur and often more difficult to resolve. They noted that study applications from experienced suicide researchers tended to deal with balancing risks and benefits and managing risks to participants more thoroughly than those that came from less experienced or novice researchers. This finding suggests that researchers unfamiliar with suicide research may underestimate their duty of care to study participants, especially when working with vulnerable population groups and in sensitive research settings, such as schools. The finding also indicates that junior researchers may benefit from support and guidance from experienced researchers when preparing suicide-related study applications.

In terms of the advice HREC members can give to researchers, the relationship between researchers and HRECs appeared to be a key factor. This has been found in prior research where research ethics committees have demonstrated a willingness to work with researchers to solve problems [10]. Researchers become more skilled at addressing the ethical issues in their study designs as they learn through the process of developing study applications, which is facilitated by ethical review. Respondents suggested that researchers who were less familiar with ethical practices in suicide-related research (for example because they are junior researchers or suicide research is not their main research field) should contact their HREC or research ethics advisor for advice in the early stages of drafting study applications. This recommendation mirrors findings from studies with suicide-related researchers, which stated that researchers may proactively seek advice from their HREC [11, 12].

Weighing risks and benefits is a key component of assessing suicide-related study applications. As in previous research, ethics committee members can see benefits as well as risks in suicide research [16]. Respondents related that personal beliefs, experiences, and feelings do play a part in the decision-making process for HRECs and these personal approaches were used in conjunction with the NHMRC National Statement on Ethical Conduct in Human Research [18]. The concept of using personal beliefs in conjunction with prescribed reviewing guidelines to assess ethics applications is important to acknowledge and has been described in other suicide-related studies where people at increased risk of suicide were recruited as study participants [6].

It is noted that Australia has a longstanding tradition of suicide research [26, 27], which may increase the likelihood of HREC members being exposed suicide-related study applications. Also, most respondents in this study had ample experience in reviewing such study applications, which may have contributed to their constructive attitudes towards suicide-related research voiced in this study.

### Strengths and limitations

The study was successful in recruiting a purposive sample of 15 respondents from HRECs within research-intense universities located in all Australian states and territories except the Northern Territory. Respondents were highly experienced ethics committee members familiar with reviewing suicide-related study applications. However, the study relied on self-report from voluntary respondents, mostly researcher members of HRECs, which may entail a selection bias. It is possible that respondents more interested in the topic were likely to self-select to participate. Also, research ethics committees in other countries may have different approaches to or may have different experiences in dealing with suicide-related study applications. Future studies in this field may involve larger samples and quantitative data to complement findings of this study.

## Conclusions

HRECs reviewing suicide-related study applications face complex ethical challenges. Reliance on ethical guidelines, personal views, and dialogue with researchers appeared to be central to the assessment of and decision-making processes regarding the study applications. Respondents were well aware of the societal importance of suicide research while being sensitive to potential risks to participants and strategies to mitigate such risks. HREC members were more likely to encounter ethical concerns in study applications from researchers with little experience in suicide research. Hence, less experienced researchers may benefit from guidance from experienced researchers and/or seek pro-actively advice from HRECs. Conversely, as some HREC members may have limited experience in dealing with suicide-related study applications, there can be merit in education and development of guidance materials for HESCs regarding suicide research. Obviously, such resources can also be beneficial for researchers. Overall, developing working relationships between HRECs and researchers will likely help both ethics committees and researchers facilitate and conduct suicide-related research.

## Supplementary Information

**Additional file 1**: COREQ Checklist

**Additional file 2**: Interview Schedule

## Data Availability

In order to protect respondent anonymity, the data (transcripts) will not be shared.

## Abbreviations

COREQ: Consolidated Criteria for Reporting Qualitative Studies
HREC: Human Research Ethics Committee
IRB: Institutional Review Board
LGBTIAQ +: Lesbian, gay, bisexual, transgender, queer or questioning, intersex and asexual people
NHMRC: National Health and Medical Research Council
REC: Research Ethics Committee
