# Supplementary material for: Ethical concerns in suicide research: thematic analysis of the views of human research ethics committees in Australia
Source: BMC Med Ethics. 2021 Apr 7;22:41. doi: 10.1186/s12910-021-00609-3 (PMC8028799; doi:10.1186/s12910-021-00609-3)
Supplement: Supplementary file 1 — Additional file 1: COREQ Checklist [file 12910_2021_609_MOESM1_ESM.docx]

Additional file 1: COREQ Checklist

| **No.** | **Item** | **Comment** |
| --- | --- | --- |
| **Domain 1: Research team and reflexivity** | | |
| 1 | Interview/facilitator | EB (page 9) |
| 2 | Credentials | EB (Ba Hons, MPH, PhD Candidate); GD (BBus, MComm, MPH, PhD Candidate); KA (PhD) |
| 3 | Occupation | EB (Academic Associate); GD (Research Assistant); KA (Research Fellow) |
| 4 | Gender | EB, GD (Female) KA (Male) |
| 5 | Experience and training | EB is an experienced qualitative interviewer. KA and JP provided supervision throughout the interview process. (pages 9-10) |
| 6 | Relationship established | No participants known to EB. GD may have known some participants due to her previous role as an ethics committee executive officer. GD was not involved in recruitment or data collection. Transcripts were deidentified prior to analysis. (page 10) |
| 7 | Participant knowledge of the interviewer | Potential participants were informed about the study by email with the participant information and consent forms attached. Potential participants know that the study was being conducted by researchers from the School of Population and Global Health at the University of Melbourne (page 9) |
| 8 | Interviewer characteristics | EB is a health sociologist and qualitative researcher with an interest in health ethics and research ethics (page 10) |
| **Domain 2: Study design** | | |
| 9 | Theoretical framework | Social constructionism/thematic analysis (pages 10-11) |
| 10 | Sampling | Purposive (pages 6-8) |
| 11 | Method of approach | Telephone, video conferencing software, and face to face (page 10) |
| 12 | Sample size | N = 15 (page 8) |
| 13 | Non-participation | 44 HRECs contacted in total (15 were ineligible, 14 did not reply) (page 8) |
| 14 | Setting of data collection | Private office (page 10) |
| 15 | Presence of non-participants | None |
| 16 | Description of sample | Human Research Ethics Committee Chairs and delegates from Australian Universities. See Table 1 (page 8) |
| 17 | Interview guide | Attached. See Additional file 2. |
| 18 | Repeat interviews | None |
| 19 | Audio/visual recording | Interviews were audio-recorded (page 10) |
| 20 | Field notes | EB recorded field notes (page 10) |
| 21 | Duration | 26 minutes to 55 minutes (page 10) |
| 22 | Data saturation | No new information at interview 15 (page 23) |
| 23 | Transcripts returned | No |
| **Domain 3: Analysis and findings** | | |
| 24 | Number of data coders | Three coders (page 11) |
| 25 | Description of the coding tree | No |
| 26 | Derivation of themes | Combined inductive and deductive approach, themes aligned with interview guide (pages 10-11) |
| 27 | Software | Microsoft word and NVivo Transcription (page 10) |
| 28 | Participant checking | No |
| 29 | Quotations presented | Yes (pages 11-20) |
| 30 | Data and findings consistent | Quotations provided to illustrate each theme |
| 31 | Clarity of major themes | Yes |
| 32 | Clarity of minor themes | Yes |
