# Supplementary material for: Ethical concerns in suicide research: thematic analysis of the views of human research ethics committees in Australia
Source: BMC Med Ethics. 2021 Apr 7;22:41. doi: 10.1186/s12910-021-00609-3 (PMC8028799; doi:10.1186/s12910-021-00609-3)
Supplement: Supplementary file 2 — Additional file 2: Interview Schedule [file 12910_2021_609_MOESM2_ESM.docx]

# Additional file 2: Interview Schedule


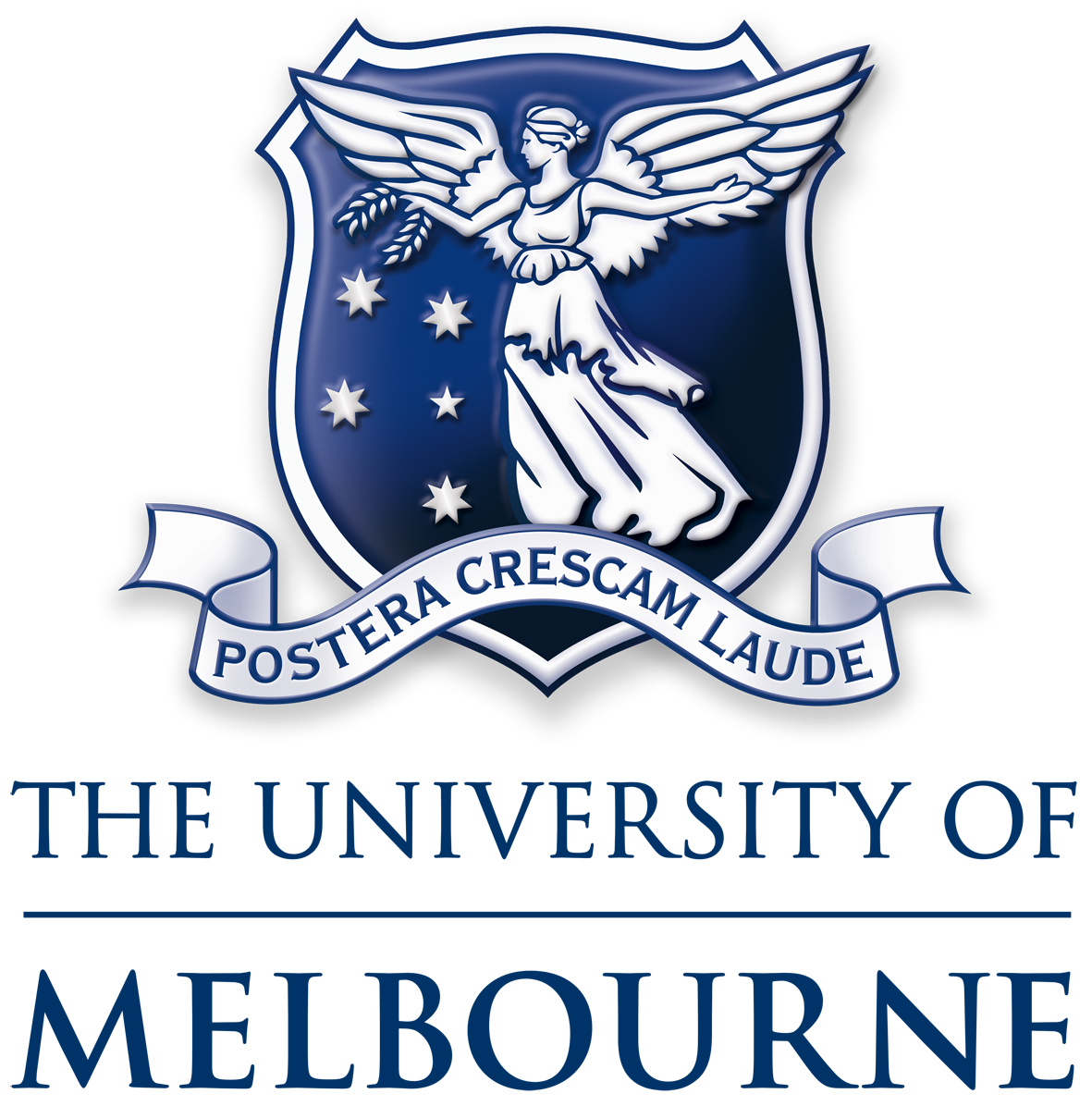


**ETHICAL ISSUES IN SUICIDE PREVENTION RESEARCH**

Thank you for agreeing to take part in this interview. We are interested in ethics committees’ experiences with considering projects involving suicide-related research.

**Can I begin by asking you a few questions about yourself?**

1. Which country do you live in?
2. What is your gender?
3. How many years of experience do you have with sitting on ethics committees?

**Next I’d like to ask you a few questions about your experiences with suicide-related project applications in your role as an ethics committee member?**

1. How many suicide-related project applications have you and your ethics committee reviewed in the last five years?
2. Thinking about all of the suicide-related project applications that you and your ethics committee have reviewed in the last five years, what percentage do you think were approved with no revisions? And what percentage do you think were approved with minor revisions? And with major revisions? And what about the percentage that were not approved?

**Now I’d like you to think about the kinds of issues that you and your ethics committee have been concerned about when reviewing the suicide-related projects that are the subject of these applications.**

1. What kind of issues have you and your ethics committee raised in relation to these projects?
2. Are these issues more common in projects with particular kinds of study designs? Or projects that employ particular data collection approaches? Or projects that focus on particular target groups? Or projects that take place in particular settings?
3. And do these issues only relate to projects that involve recruiting participants? Or are there other, more generic, issues?
4. What sorts of recommendations have you and your ethics committee made to researchers with respect to resolving these issues?
5. How would you describe the approach that you and your ethics committee take to balancing risks and benefits in suicide-related projects? What sort of things get taken into consideration in striking this balance?
6. When you and your ethics committee have raised concerns with researchers about suicide-related projects, what sort of responses have you received?
7. As a general rule, when researchers have addressed these concerns, do you think that this has had a positive impact on the project? Are there occasions when it may have had a negative impact?

**And one final general question …**

1. Based on your overall experience with reviewing ethics applications for suicide-related projects, do you have any advice for researchers?

**Thank you for taking the time to participate in the interview**
